# Tomatidine Exposure During Early Myogenesis Promotes Hypertrophy of C2C12 Muscle Cells Through Increased Nuclei Accretion

**DOI:** 10.1002/cbin.70224

**Published:** 2026-10-08

**Authors:** Marissa K. Caldow, Kristy Swiderski, John H. V. Nguyen, Emily S. Haber, Kyla J. H. Fritz, Gordon S. Lynch

**Affiliations:** ^1^ Centre for Muscle Research, Department of Anatomy and Physiology School of Biomedical Sciences, Faculty of Medicine, Dentistry and Health Sciences, The University of Melbourne Victoria Australia

**Keywords:** bioactives, hypertrophy, myogenesis, skeletal muscle, tomatidine

## Abstract

During myogenesis or myofiber regeneration, a proliferating muscle cell must leave the cell cycle, commit to differentiate, and fuse to neighboring myoblasts to create multinucleated myotubes, and eventually mature myofibers. These stages can be manipulated by exogenous factors including signaling molecules, mechanical cues, and nutrient supplementation. Supplementation with bioactives, such as tomatidine, can improve aspects of skeletal muscle health when evaluated using in vitro and in vivo models. In this study, a phenotypic characterization of the effects of tomatidine supplementation during proliferation, early maturation and differentiation, was undertaken in C2C12 muscle cells, cultured in growth medium, or alternatively induced to differentiate in medium supplemented with 10 µM tomatidine, for up to 4 days. Real‐time impedance assays indicated tomatidine slowed C2C12 proliferation after 3 days of supplementation. Tomatidine supplementation from the onset of differentiation induced myotube hypertrophy within the first 48 h of maturation. Tomatidine‐induced hypertrophy was driven by increased myonuclei fusion in developing myotubes with a concomitant increase in protein synthesis. Taken together, tomatidine's growth promoting effects on skeletal muscle cells identify its therapeutic potential for enhancing muscle regeneration and repair, by mechanisms that improve myofiber maturation and function.

**Abbreviations**
4EBP1: eukaryotic translation initiation factor 4E binding protein 1
ANOVA: analysis of variance
DAPI: 4′,6‐diamidino‐2‐phenylindole
DM: differentiation media
DMEM: Dulbecco's Modified Eagle Medium
DMSO: dimethyl sulfoxide
GM: growth media
JAK: Janus Kinase
mTORC1: mechanistic target of rapamycin complex 1
MuSCs: muscle stem cells
MyoD: myogenic differentiation 1
p70S6K: ribosomal protein S6 kinase
PBS: phosphate buffered saline
PVDF: polyvinylidene difluoride
RTCA: Real‐Time Cell Analyzer
S6: Ribosomal Protein S6
SD: standard deviation
SDS‐PAGE: sodium dodecyl sulfate–polyacrylamide gel electrophoresis
STAT3: signal transducer and activator of transcription 3
TOM: tomatidine

## 1 Introduction

Skeletal muscle has a distinct capacity for regeneration, largely attributed to its intrinsic population of quiescent muscle stem cells (MuSCs) located beneath the basal lamina of muscle fibers. In response to injury or other external perturbing stimuli, MuSCs become activated, and enter the cell cycle to undergo muscle cell development (myogenesis). Activated MuSCs differentiate into myoblasts that proliferate and subsequently fuse with one another to form multinucleated myotubes, or fuse with existing myofibers to support hypertrophy and myofiber repair (Dumont et al. 2015). MuSC activation, successful transition through myogenesis and ultimately muscle repair, is tightly regulated by a precisely timed inflammatory response involving local and circulating immune cells. Disruption of this crosstalk can lead to chronic inflammation and impaired regeneration, contributing to pathological conditions (Tidball 2017).

There is significant interest in nutritional strategies to modulate or enhance skeletal muscle regeneration and promote muscle health, particularly bioactive peptides that can manipulate biological signaling in skeletal muscle for commercial, therapeutic and agricultural applications, including cellular agriculture for the production of cultivated meat and seafood (Lynch and Koopman 2019). Plant‐derived bioactives have the potential to steer muscle cell development for novel cell therapies in regenerative medicine and for applications relevant to cellular agriculture. Among the most significant challenges for cellular agriculture are the optimization of myogenesis and maturation of muscle cell phenotype, events crucial for the scaled manufacture of cultivated meat and seafood products (Trautmann et al. 2025; Zhen et al. 2026). Enhancing muscle cell maturation through fusion and growth has proved challenging with standard cell culture but modulating nutrients in serum or ‘nutritive steering', is a novel approach for stimulating muscle protein accrual, promoting maturation, and growing muscle to scale, appropriate for food production.

Nutritional supplementation is advantageous since many compounds have established safety and pharmacokinetic profiles facilitating rapid translation (Fatima et al. 2025). Among these, plant‐derived bioactive compounds, including flavonoids, carotenoids, and alkaloids, are widely accessible for research and therapeutic application (Zaky et al. 2024). Tomatidine, a natural small molecule alkaloid found in unripe tomatoes, exhibits diverse pharmacologic properties (Bailly 2021). Emerging evidence indicates tomatidine has beneficial effects on skeletal muscle mass and health. For example, in healthy mice, tomatidine supplementation increased muscle mass, myofiber size, and exercise capacity, while attenuating muscle wasting during caloric restriction and limb immobilization (Dyle et al. 2014). Mechanistically, tomatidine activates mTORC1 signaling promoting protein synthesis and hypertrophy in differentiated myotubes (Dyle et al. 2014). In addition to its potential anabolic effects, tomatidine attenuates inflammatory responses to exercise‐like stimulation in human donor‐derived skeletal muscle myo‐bundles, suggesting a potential role in modulating the inflammatory environment that critically regulates muscle regeneration (Parafati et al. 2025).

Despite these advances, whether tomatidine can exert effects during the early stages of myogenesis is poorly understood. Given the importance of early differentiation and fusion events in skeletal muscle regeneration, it is important to determine whether tomatidine can elicit growth‐promoting effects during these early stages of muscle fiber formation. To address this shortfall in knowledge, the aim of this study was to characterize how tomatidine supplementation affects proliferation, differentiation, and maturation of C2C12 muscle cells. To this end, cells were cultured in growth media or induced to differentiate in media supplemented with 10 µM tomatidine, for up to 4 days. Morphological and functional outcomes, including myotube size and myoblast–myotube fusion, were assessed over time. The findings reveal how tomatidine can enhance myogenic differentiation and promote myotube hypertrophy, identifying its therapeutic potential for improving skeletal muscle regeneration.

## 2 Materials and Methods

### 2.1 Cell Culture

Murine C2C12 myoblasts (ATCC Cryosite distribution, NSW, Australia) were cultured in growth media [GM, DMEM (#11965; ThermoFisher Scientific, Waltham, MA, USA) supplemented with 10% (v/v) fetal bovine serum and 1% (v/v) antibiotic solution (100 unit/ml penicillin/streptomycin) (Life Technologies, ThermoFisher Scientific)] at 37°C in an atmosphere of 5% CO_2_. Upon confluency, the media was changed to differentiation media [DM, DMEM (#11965; ThermoFisher Scientific) supplemented with 2% (v/v) horse serum, 1% antibiotic solution (Life Technologies, ThermoFisher Scientific)] for 4 days to promote formation of mature multinucleated myotubes.

### 2.2 Tomatidine Exposure During Myogenesis

Tomatidine (as tomatidine hydrochloride, #T2909, Sigma‐Aldrich, NSW, Australia) was dissolved in dimethyl sulfoxide (DMSO). For proliferation experiments, cells were grown in Petri dishes until required for seeding into E‐Plate 16‐well gold‐electrode sensor plates for use in the xCELLigence Real‐Time Cell Analyzer (RTCA) DP Instrument (Agilent Technologies Inc., San Diego, CA).

For differentiation experiments, C2C12 cells were grown in GM in 24‐well plates, until the myoblasts reached 95% confluence. Cells were rinsed with phosphate buffered saline (PBS) and media was replaced with treatment medium prepared in DM (10 µM tomatidine, or an equal amount of DMSO as the vehicle control) [Day 0 of differentiation, D0]. At day two [D2], cells were rinsed once in PBS before refreshing the treatments or replacing treatment media with vehicle in washout experiments. On day 4 [D4] cells were prepared for endpoint analyses. Brightfield images were captured daily at 10× magnification using a Zeiss Primovert Microscope with a Zeiss AxioCam ERC 5 s Rev 2.0 camera (Carl Zeiss AG, Germany), to visualize daily morphological changes (Figure S1, Supplemental Material).

To determine whether a shorter duration of tomatidine treatment affected differentiation, differentiating myoblasts were supplemented with tomatidine for the first 2 days of differentiation only (D0‐D2; TOM > DMSO) and then returned to vehicle supplemented differentiation media for a further 2 days. The treatment protocol was also inverted to Day 2–4 of differentiation (DMSO > TOM), where cells were differentiated in DMSO for the first 2 days of differentiation, then swapped to tomatidine supplementation for the next 2 days.

### 2.3 Analysis of C2C12 Proliferation via Impedance Measurements

Real‐time, label‐free, electric impedance measurements were performed using the xCELLigence Real‐Time Cell Analyzer (RTCA) DP Instrument. Prior to seeding cells, background impedance was recorded after adding 100 µL GM to each well (Sweep interval = 2 min). Cells (1000) were seeded into 15 wells in 100 µL GM. One well remained cell‐free with a total of 200 µL of GM, to serve as a blank. Plates were incubated at room temperature for 30 min to allow cells to equilibrate, then inserted into the RTCA cradle for continuous impedance monitoring (Sweep interval = 20 min).

After 24 h, the instrument was paused, the plate removed and media replaced with 200 µL GM supplemented with 10 µM tomatidine, or an equal amount of DMSO as the vehicle control. Plates were incubated at room temperature for 30 min to allow cells to equilibrate then inserted into the RTCA cradle and impedance monitoring resumed. This process was repeated after 48 h in treatment media to allow for media refreshment.

Impedance readings were collected every 20 min for a total of 100 h. Cell Index data generated by the xCELLigence during the 72 h treatment period was normalized to the time when treatment media was added (T0).

### 2.4 Myotube Diameter and Nuclei Counting for Fusion Index and Differentiation Index

Cells were cultured in 24‐well plates and immunostained for myosin (MF20), as described previously (Caldow et al. 2019). Cells were imaged within the tissue culture plate using an automated Perkin Elmer Operetta High Content Imaging system (PerkinElmer, Waltham, MA, USA), with a 20× LWD objective, controlled by Harmony High Content and Imaging Analysis Software (Rel. 4.1, Perkin Elmer). Six images were acquired in each well from pre‐defined locations (*n* = 6 fields of view).

Myotube diameter in cells positive for MF20 (MF20^+^ cell) was measured using AxioVision software (AxioVision AC Rel. 4.8.2, Carl Zeiss Imaging Solutions, Wrek, Göttingen; Germany). An average of 80 myotubes were measured per well and the average diameter of each well was used for statistical analysis.

The number of multinucleated muscle cells (MF20^+^ cell with more than two nuclei) and nuclei (via DAPI counterstaining) within each six fields of view, per well, was performed using the Cell Counter plugin in Fiji (ImageJ) (Schneider et al. 2012). The total number of nuclei was counted, as well as the number of nuclei within each MF20^+^ cell. The percentage of nuclei within fibers, relative to the total nuclei was designated as the “Fusion Index”. The number of nuclei in MF20^+^ multinucleated cells, relative to the total number of MF20^+^ multinucleated cells was designated as the “Differentiation Index”.

### 2.5 Live Cell Imaging

Cells were cultured in 24‐well plates, as described. After treatments were refreshed on D2, all wells without cells were filled with PBS, and the plate sealed with a gas permeable film (Axygen, Corning, NY, USA) followed by the plate lid. Cells were placed into the Perkin Elmer Operetta High Content Imaging system at 37 ^o^C in 5% CO_2_ for 46 h. Images were captured every hour from 33 pre‐defined locations within each well, using a 10× LWD objective. At the end of the 46 h treatment period, cells were removed from the Operetta and images were processed using Harmony 4.1 software and Image J (Schneider et al. 2012).

### 2.6 Protein Synthesis and Immunoblotting

Myotubes were grown in 24‐well plates and treated as described. SUnSET methodology was used to determine the rate of protein synthesis (Goodman and Hornberger 2013; Ham et al. 2014). Briefly, puromycin (Sigma‐Aldrich) was administered to the media at a final concentration of 1 µM exactly 30 min before cells were collected at D0, 1 h, D2, D2 + 1 h (49 h) and D4 of differentiation. Samples for 1 and 49 h were collected following application/refreshment of differentiation media. Cells were lysed in 150 µL ice‐cold RIPA buffer (Merck Millipore, VIC, Australia) including protease and phosphatase inhibitor cocktails (Sigma‐Aldrich). Lysates were prepared and used for immunoblotting, and gels run, as described previously (Caldow et al. 2019). Proteins were transferred to 0.45 µm PVDF membrane via wet transfer (with 20% methanol) and images captured. Membranes were handled as described (Caldow et al. 2019). Proteins were visualized using the ChemiDoc™ imaging system (Bio‐Rad). Blots were quantified using ImageLab® software (Bio‐Rad) and normalized to total protein as quantified from the Stain Free blot imaging. Anti‐puromycin was purchased from Millipore (#MABE343, Kilsyth, Victoria, Australia). pSTAT3 Tyr705 (#9131), STAT3 (#4904), pp70S6K Thr389 (#9205), p70S6K (#9202), pS6 Ser240/244 (#2215), S6 (#2217), p4BEP1 Ser65 (#9451), 4EBP1 (#9644) were purchased from Cell Signaling (Danvers, MA, USA).

### 2.7 Statistical Analysis

All values were expressed as mean ± SD, representing data from three experimental replicates. Data were processed using GraphPad Prism 10.0 software. A two‐way ANOVA (time and treatment) with a Bonferroni post hoc test was used to compare between groups for cell proliferation analysis via the xCELLigence Real‐Time Cell Analyzer. T‐tests or one‐way ANOVAs with a Dunnett's post hoc test were used to compare groups against DMSO control cells for all other analysis. If needed, a mixed‐effects analysis was performed. For transparency, both significant differences (*p* < 0.05) and trends (*p* < 0.1) are reported, where appropriate.

## 3 Results

### 3.1 Tomatidine Reduces C2C12 Myoblast Proliferation

To examine the effect of tomatidine supplementation on myoblast proliferation, C2C12 cells were exposed to 10 µM tomatidine during proliferation and cultured in the xCELLigence Real‐Time Cell Analyzer (RTCA) DP Instrument (Figure 1A). After 36 h of tomatidine supplementation, Cell Index, calculated from impedance data, was reduced compared with vehicle treated cells (−52%, *p* < 0.01, Figure 1B‐C), which was maintained for the remainder of the treatment period (72 h). Consistent with this finding, tomatidine supplemented cells exhibited a strong trend toward an increased mean doubling time (*p* = 0.052, Figure 1D). Taken together, these data suggest that tomatidine reduces myoblast proliferation.

**Figure 1:**
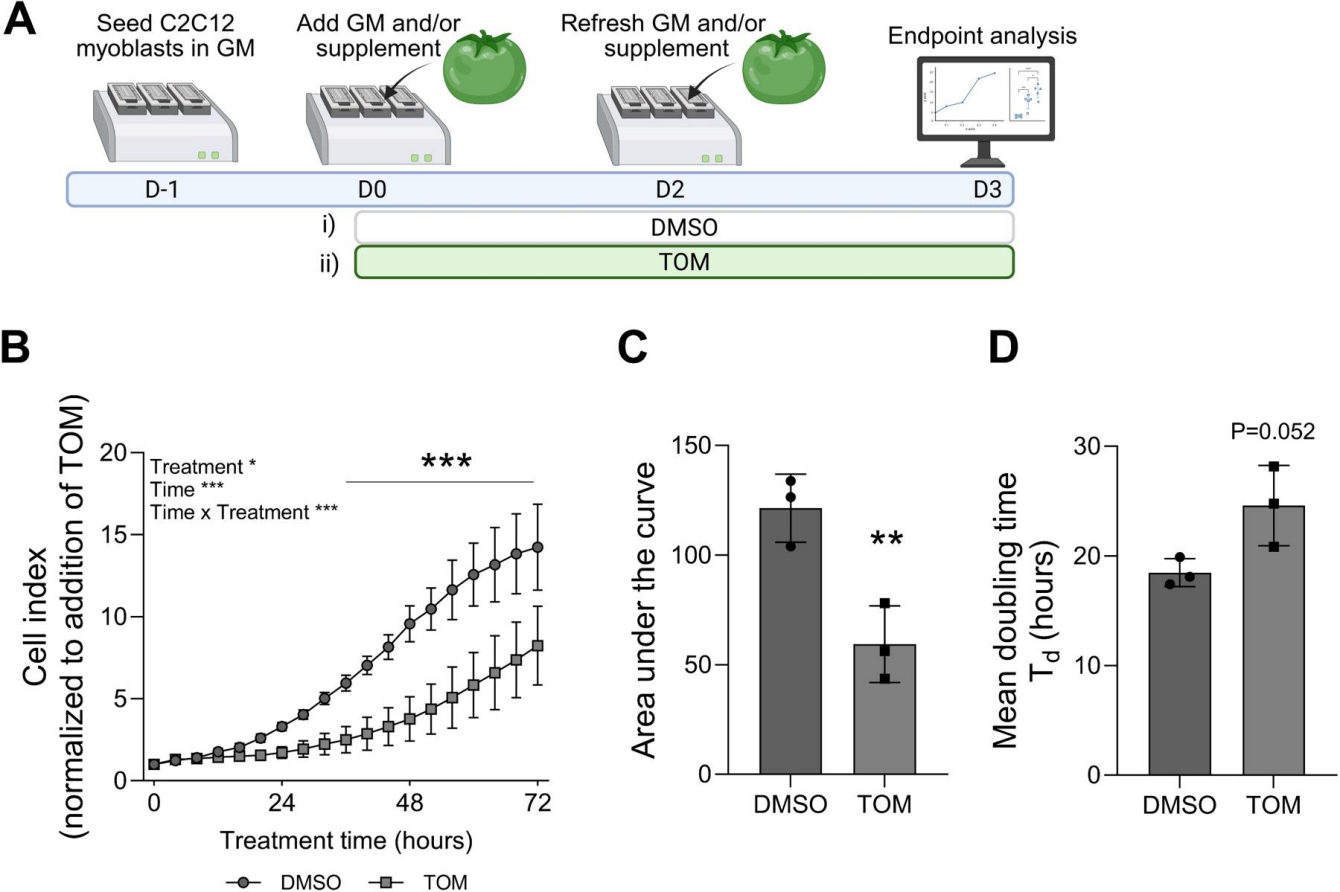
Exposure of muscle cells to tomatidine during proliferation reduces C2C12 myoblast proliferation. C2C12 cells were grown for 3 days in standard growth media containing vehicle control (DMSO) or media supplemented with 10 µM tomatidine, in the xCELLigence Instrument. (A) Schematic of experimental timeline. Created in BioRender. Caldow, M. (2026) https://BioRender.com/paic1qg. (B) Growth curve of Cell Index over 72 h of treatment time, calculated via electric impedance measurements on the xCELLigence Instrument. (C) Area under the curve of Cell Index data. (D) Mean doubling time (Td), calculated from electric impedance measurements on the xCELLigence. Data are presented as mean ± SD (*n* = 3 experiments). Significantly different to DMSO, **p < 0.01, ***p < 0.001. DMSO, vehicle supplemented control cells. TOM, cells supplemented with 10 µM tomatidine.

### 3.2 Exposure of Muscle Cells to Tomatidine During Early Differentiation Promotes Hypertrophy

To examine the effect of tomatidine supplementation on myoblast differentiation, C2C12 myoblasts were exposed to 10 µM tomatidine from the onset of differentiation [D0] for up to 4 days (Figures 2, 3). After 2 days of differentiation [D2], mature myotubes were already present, and were larger after supplementation with tomatidine compared with control cells (9%, *p* < 0.01, Figure 2B,C). This effect persisted through to day 4 of differentiation (D4), with tomatidine myotubes remaining larger than controls (17%, *p* 0.01, Figure 3B,C).

**Figure 2:**
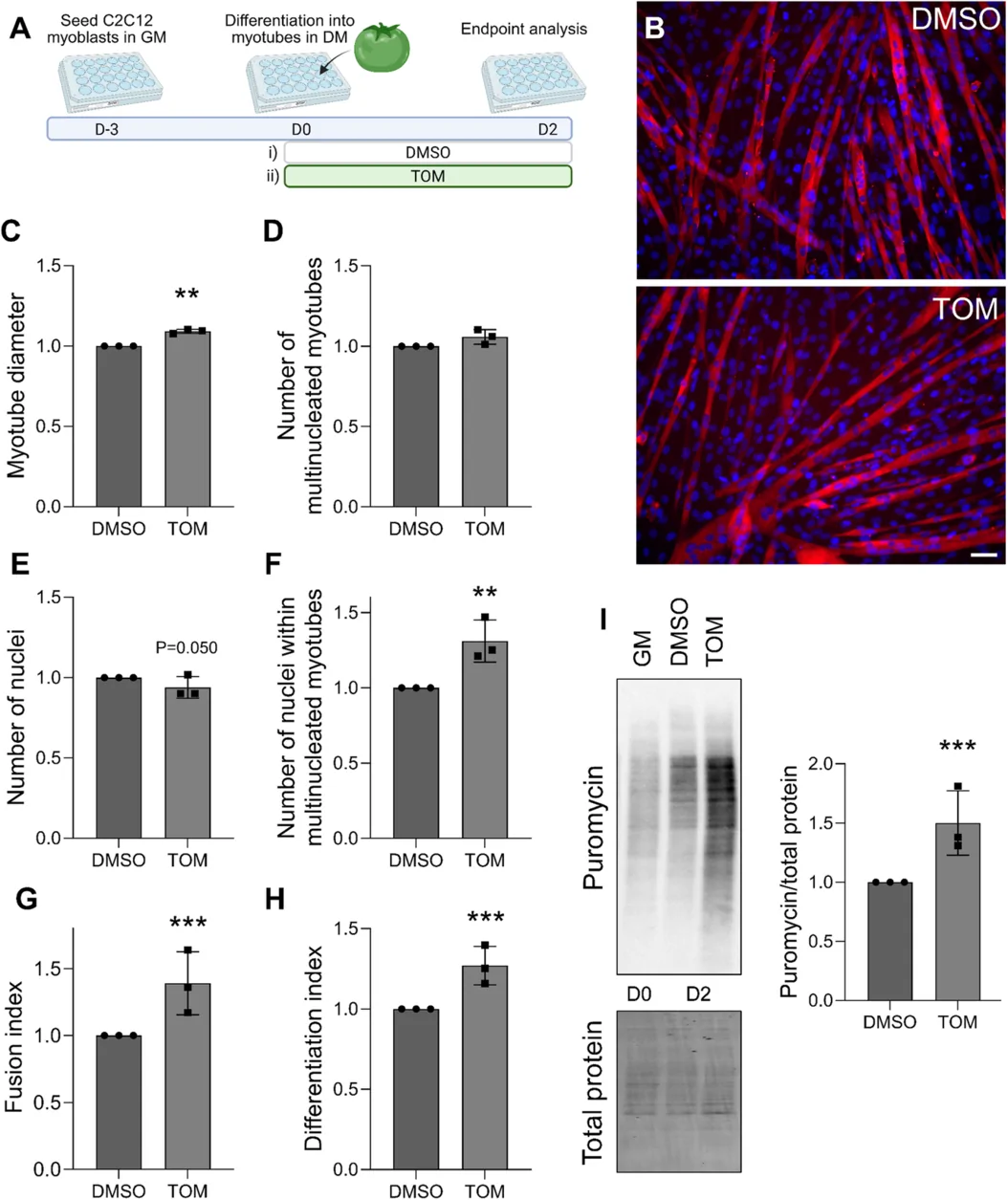
Muscle cells supplemented with tomatidine during early differentiation exhibit hypertrophy and increased cell fusion. C2C12 cells were differentiated for 2 days in standard differentiation media containing vehicle control (DMSO) or media supplemented with 10 µM tomatidine from the onset of differentiation. (A) Schematic of experimental timeline. Created in BioRender. Caldow, M. (2026) https://BioRender.com/8nz1y75. (B) Representative images of C2C12 cells differentiated for 2 days in standard differentiation media containing vehicle control (DMSO) or media supplemented with 10 µM tomatidine from the onset of differentiation. Cells were immunostained for myosin (red) and DAPI (blue, nuclei). (C) Myotube diameter. (D) Number of multinucleated MF20+ cells. (E) Number of nuclei. (F) Number of nuclei within multinucleated MF20+ cells. (G) Fusion index. (H) Differentiation index. (I) Puromycin protein abundance and representative immunoblot. Data are presented as mean ± SD (*n* = 3 experiments), normalized to DMSO. Significantly different to DMSO, **p < 0.01, ***p < 0.001. GM, growth media before differentiation media was applied. DMSO, vehicle treated control cells. TOM, cells supplemented with 10 µM tomatidine. Scale bar represents 50 µm.

**Figure 3:**
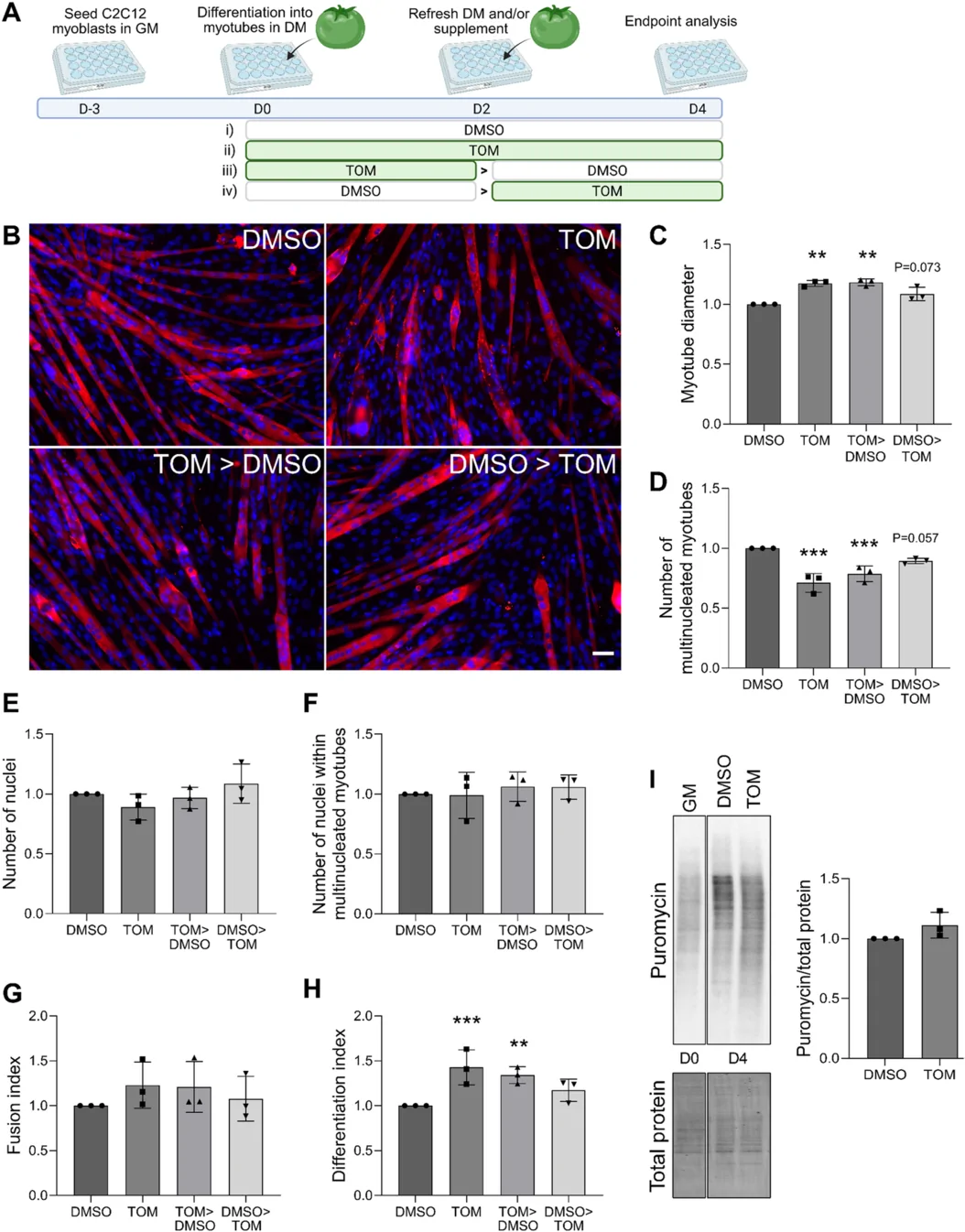
The growth promoting effects of tomatidine supplementation on C2C12 myotubes are maintained even after returning to differentiation media. (A) C2C12 cells were differentiated for 4 days in standard differentiation media containing vehicle control (DMSO) or media supplemented with 10 µM tomatidine from the onset of differentiation. Cells were also supplemented with 10 µM tomatidine from differentiation Day 0 to Day 2, then returned to vehicle treatment media from Day 2 to Day 4 (TOM > DMSO). Or cells were supplemented with tomatidine from differentiation day 2 to day 4 only (DMSO > TOM). Created in BioRender. Caldow, M. (2026) https://BioRender.com/foud55w. (B) Representative images of cells immuno‐stained for myosin (red) and DAPI (blue, nuclei). (C) Myotube diameter. (D) Number of multinucleated MF20+ cells. (E) Number of nuclei. (F) Number of nuclei within multinucleated MF20+ cells. (G) Fusion index. (H) Differentiation index. (I) Puromycin protein abundance and representative immunoblot. Data are presented as mean ± SD (*n* = 3 experiments), normalized to DMSO. Significantly different to DMSO, **p < 0.01, ***p < 0.001. Scale bar represents 50 µm. GM, growth media before differentiation media was applied. DMSO, vehicle treated control cells. TOM, cells supplemented with 10 µM tomatidine.

To determine whether the early effects of tomatidine during differentiation reflected a “priming mechanism” that promotes hypertrophy, cells were supplemented with 10 µM tomatidine from D0‐D2 and then returned to vehicle treatment media from D2 to D4 (TOM > DMSO; Figure 3A). Similar to the findings with continuous tomatidine supplementation from D0‐D4, cells maintained their hypertrophic trajectory, even when tomatidine was removed from the media during the second 48‐h period (18% larger compared to control, *p* < 0.01, Figure 3B,C). By comparison, cells supplemented with tomatidine only during the later phase (D2–D4, DMSO > TOM) trended to be larger than control cells (9%) (*p* = 0.073, Figure 3B,C).

### 3.3 Tomatidine‐Induced Hypertrophy of Muscle Cells During Early Differentiation Is Driven by Greater Cell Fusion

To determine how tomatidine promotes hypertrophy during differentiation, we quantified myogenic progression by assessing the number of multinucleated MF20^+^ cells, the number of nuclei within MF20^+^ multinucleated cells, and total nuclei content.

At D2, tomatidine supplementation did not alter the number of MF20^+^ multinucleated cells compared with control conditions (Figure 2D), but there was a reduction in total nuclei (−6%, *p* = 0.050, Figure 2E), accompanied by an increase in the number of nuclei within MF20^+^ cells (31%, *p* < 0.01, Figure 2F). Consistent with enhanced myogenic maturation, both fusion index (39%, *p* < 0.001, Figure 2G) and differentiation index (27%, *p* < 0.001, Figure 2H) were increased after tomatidine supplementation.

By D4, tomatidine supplementation displayed a reduction in the number of MF20^+^ multinucleated cells (−29%, *p* < 0.001, Figure 3D), although there were no differences in the total number of nuclei, number of nuclei per myotube, or the fusion index (Figure 3E–G). In contrast, differentiation index remained elevated, indicating a greater proportion of nuclei within MF20^+^ cells (43%, *p* < 0.001, Figure 3H).

A similar phenotype was observed after early, transient tomatidine supplementation (D0–D2, TOM > DMSO). There were fewer MF20^+^ multinucleated cells (−21%, *p* < 0.001, Figure 3C), with no changes in number of nuclei within these cells or total number of nuclei, or fusion index (Figure 3D–F), but an increased differentiation index (34%, *p* < 0.01, Figure 3G). In contrast, tomatidine supplementation during the later phase (D2‐D4, DMSO > TOM), reduced the number of MF20^+^ multinucleated cells (−11%, *p* = 0.057, Figure 3C), with no effects on the number of nuclei within these cells, total number of nuclei, fusion index, or differentiation index (Figure 3D–G).

Myotube‐myotube fusion was observed in tomatidine treated cells using live cell imaging (Figure 4A). From D2‐D4, vehicle supplemented cells generally exhibited linear growth and fusion with neighboring myoblasts, (DMSO, Figure 4B, Ci; Video S1, Supplemental material). In contrast, developing myotubes supplemented with tomatidine, have increased myonuclei fusion to neighboring myotubes (TOM, Figure 4B, Cii) and displayed highly dynamic non‐linear movements, where large myotubes migrated towards one another, followed by direct myotube‐myotube fusion (TOM, Figure 4B, Ciii; Video S2, Supplemental material). These dynamic fusion events likely contributed to the reduced number of myotubes present, and the increased differentiation index observed with tomatidine supplementation (Figures 3C, 3G).

**Figure 4:**
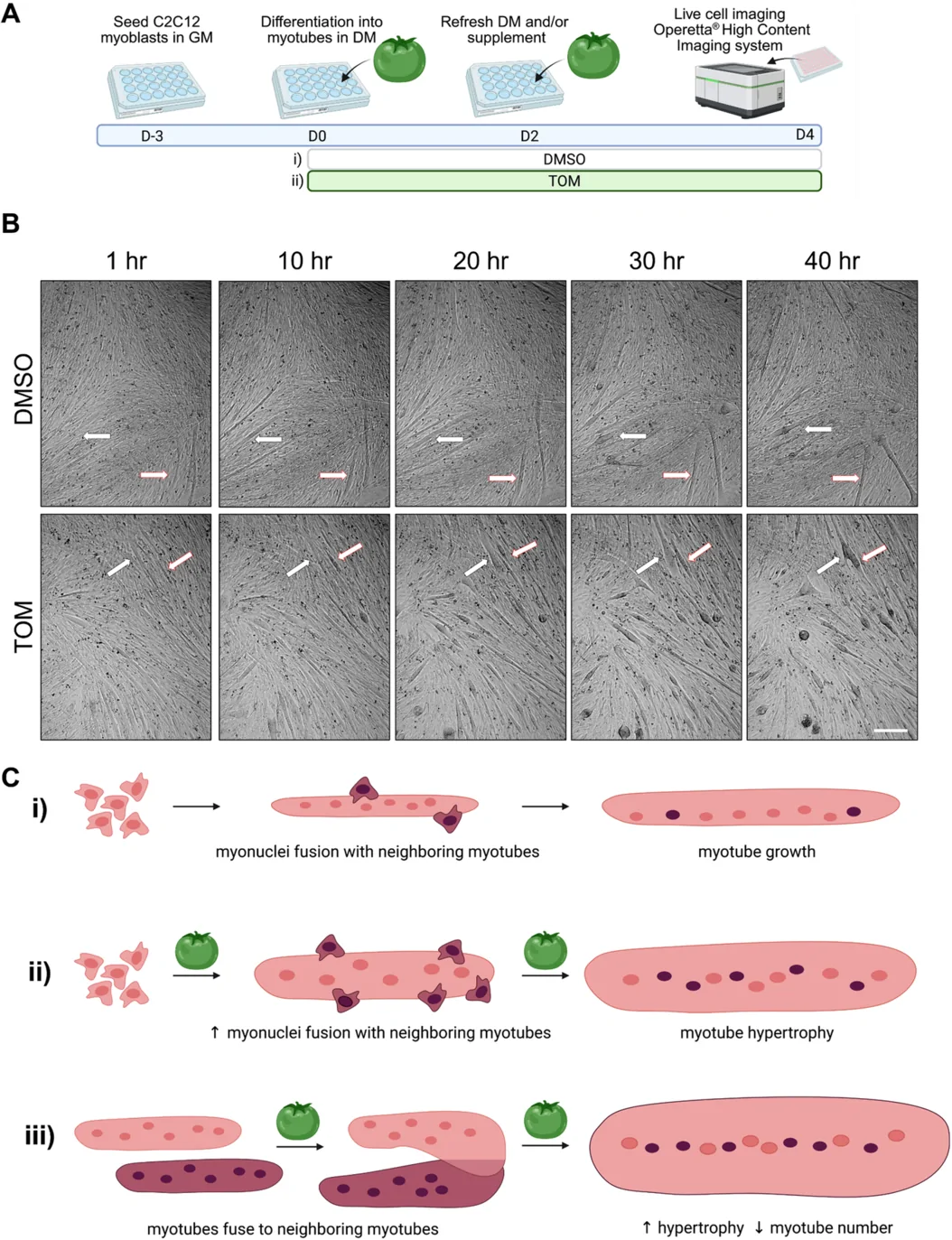
Tomatidine supplementation promotes myotube‐myotube fusion. C2C12 cells were differentiated for 4 days in standard differentiation media containing vehicle control (DMSO) or media supplemented with 10 µM tomatidine from the onset of differentiation. (A) Live cell imaging was performed from D2 to D4 of differentiation. Images were captured each hour, across 45 h. Figure created with BioRender. Caldow, M. (2026) https://BioRender.com/u1n0wm7. (B) Representative brightfield images of cells over time. (C) Schematic explaining differentiation under normal conditions (i), effects of tomatidine supplementation to promote hypertrophy via increased myonuclei‐myotube fusion (ii) or (iii) myotube‐myotube fusion. Created in BioRender. Caldow, M. (2026) https://BioRender.com/hpcc0s9. Scale bar represents 200 µm. DMSO, vehicle treated control cells. TOM, cells supplemented with 10 µM tomatidine.

### 3.4 Tomatidine‐Induced Hypertrophy Is Accompanied by Increased Protein Synthesis

To establish whether tomatidine‐induced hypertrophy during early maturation was accompanied by an increase in protein synthesis, changes in puromycin abundance were assessed using the SUnSET methodology. Within 1 h of inducing differentiation in the presence of tomatidine, puromycin abundance was significantly increased (*p* < 0.001, Figure S2, Supplemental material). This elevation was maintained after 48 h of differentiation, with puromycin abundance remaining higher in tomatidine supplemented cells (50%, *p* < 0.001, Figure 2I). By D4, however, there was no difference in puromycin abundance between DMSO and tomatidine supplemented cells (Figure 3I). This occurred without simultaneous changes to STAT3, p70S6K, S6 or 4EBP1 phosphorylation (Figure S3, Supplemental material).

## 4 Discussion

Tomatidine, a natural component found in unripe, green tomatoes, possesses many pharmacologic properties (Bailly 2021). While the molecular targets of tomatidine in humans have not been elucidated completely, several studies have identified potential health benefits including anti‐inflammatory (Chiu and Lin 2008; Costa et al. 2026), neuroprotective (Costa et al. 2026; Wang et al. 2021), anti‐cancer effects (Fujimaki et al. 2022; Mukherjee et al. 2023), and senotherapeutic actions that reduce cellular senescence and improve cognitive function in aged mouse models (Costa et al. 2026).

Accumulating evidence from studies in skeletal muscle demonstrates that tomatidine promotes muscle growth and inhibits muscle atrophy by stimulating protein synthesis and mitochondrial function (Dyle et al. 2014; Ebert et al. 2015). In addition to these anabolic effects, tomatidine attenuates inflammation in human donor‐derived skeletal muscle myo‐bundles, partly through modulation of cytokine‐responsive pathways such as JAK/STAT signaling (Parafati et al. 2025). However, previous studies have largely focused on animal models or mature cultured myotubes (Dyle et al. 2014; Ebert et al. 2015; Parafati et al. 2025), and it remains unclear whether tomatidine can influence myotube maturation, differentiation, or growth during early myogenesis. The present study revealed that tomatidine can influence multiple stages of myogenesis in C2C12 cells, including proliferation, early maturation, and differentiation. Thus, tomatidine supplementation has potential for steering muscle cell development during these events, relevant to applications in regenerative medicine and cellular agriculture. To the best of our knowledge, this study is the first to demonstrate that tomatidine modulates muscle cell proliferation and induces myotube hypertrophy through increased myoblast‐myotube fusion.

Previous studies examining tomatidine in C2C12 myoblasts examined its effects on apoptosis and inflammation (Song et al. 2018), while its influence on early myogenic events and proliferation dynamics had not yet been assessed. Our findings revealed that tomatidine supplementation during proliferation reduced Cell Index (as measured via the xCELLigence^TM^ platform) and increased doubling time, indicating tomatidine attenuated cell proliferation. These effects are consistent with the anti‐proliferative actions of tomatidine in other cell types, such as pancreatic cancer cells (Mukherjee et al. 2023), gastric cancer‐derived 85As2 cells (Fujimaki et al. 2022), or fibroblast‐like synoviocytes (Yu et al. 2021). Given the importance of MuSC and myoblast proliferation during the early stages of skeletal muscle regeneration, these findings suggest tomatidine supplementation may exert stage‐specific effects, whereby early exposure could compromise optimal regenerative capacity.

It is important to acknowledge that assessments of proliferation in the present study were derived from xCELLigence impedance measurements, including Cell Index and doubling time, which reflect a combination of cell number, adhesion, morphology, and spreading, rather than cell division alone. Although the observed reduction in Cell Index and increased doubling time after tomatidine treatment are consistent with attenuated proliferation, the tomatidine‐induced changes in cell adhesion or cytoskeletal organization may also contribute to the impedance phenotype. Therefore, these findings should be interpreted as evidence of altered impedance characteristics consistent with reduced proliferative status, rather than as a direct measurement of cell proliferation. Future studies incorporating direct measures of cell proliferation, such as EdU incorporation, Ki‐67 staining, or DNA synthesis assays, will be important to confirm these effects.

During myogenesis and myofiber regeneration, proliferating muscle cells must exit the cell cycle, commit to differentiation, and fuse with neighboring myoblasts to create multinucleated myotubes and ultimately, mature myofibers. Differentiation is induced *in vitro* by exposing proliferating myoblasts to low serum conditions. Although this process is tightly regulated, it occurs asynchronously due to differences in cell cycle status and local intracellular signaling. To understand how tomatidine influences this transition and development process, we assessed its effects from the onset of differentiation, a stage not investigated previously. Prior studies have exposed formed myotubes (at day 7 of differentiation) to tomatidine for 48 h (Dyle et al. 2014), showing mTORC1‐dependent hypertrophy, but had not assessed changes in myonuclei number or fusion events at this stage. Our findings extend this knowledge by showing tomatidine promotes hypertrophy during the early stages of differentiation. Notably this early hypertrophic response occurred alongside a reduction in myonuclei number compared with control conditions, during the first 48 h of differentiation. It is possible tomatidine suppresses the final round of myoblast proliferation that typically occurs during the transition from growth media to differentiation media, as cells adapt to the altered metabolic and signaling cues. Alternatively, tomatidine may accelerate the exit from the cell cycle and commitment to differentiation, thereby biasing cells towards fusion rather than proliferation.

In line with this, despite total myonuclei number being reduced, tomatidine supplemented cells displayed a greater number of myonuclei within myotubes, alongside increased myotube diameter. Interestingly, prolonged tomatidine supplementation resulted in fewer myotubes overall, despite this coinciding with a higher differentiation index, suggesting enhanced myotube‐myotube fusion. Together, these findings suggest tomatidine promotes hypertrophy by supporting myonuclei accretion and secondary fusion events leading to the formation of fewer but larger, myotubes with greater myonuclei content; features associated with improved muscle regenerative capacity.

Consistent with these structural adaptations and the observed hypertrophic phenotype, tomatidine supplementation also transiently increased protein synthesis during early differentiation, by an increased abundance of puromycin. This was evident during the initial stages of differentiation and sustained during early myotube formation, aligning with the increases in myotube size and fusion capacity. These findings are consistent with previous reports of tomatidine stimulating protein synthesis in differentiated myotubes (Dyle et al. 2014). Our data extend these observations, demonstrating the anabolic effect occurs much earlier during myogenesis, suggesting tomatidine enhances translational activity during a critical window of myotube formation. Notably, this increase in protein synthesis was not maintained at later stages of differentiation, indicating tomatidine may exert temporally restricted anabolic effects that are most pronounced during early myogenic progression, and may contribute to the sustained hypertrophic phenotype after early exposure.

To determine whether these hypertrophic effects required sustained tomatidine exposure, we reduced the exposure period from the initial 4‐day differentiation protocol. When differentiating myoblasts were supplemented with tomatidine only during the first 2 days of differentiation (D0‐D2; TOM > DMSO), then returned to vehicle supplemented media, myotubes maintained their hypertrophic trajectory comparable to that with continuous tomatidine supplementation. Across all parameters assessed, cells exposed to early or sustained tomatidine supplementation followed comparable patterns, suggesting early tomatidine supplementation exerts a carry‐over effect that may prime muscle cells for hypertrophy by enhancing fusion capacity. In contrast, tomatidine supplementation restricted to the later stages of differentiation (D2–D4; DMSO > TOM) did not significantly increase myotube width by the D4 endpoint and resulted in fewer multinucleated myotubes than in control conditions. Together, these findings highlight a critical early window during which tomatidine exerts its pro‐myogenic effects, although future studies will need to examine myotube morphology over extended periods of differentiation (e.g., beyond Day 6) to determine whether delayed or secondary fusion events contribute to later hypertrophic outcomes.

Although the mechanisms remain to be fully elucidated, several pathways may underlie the pro‐myogenic effects of tomatidine observed in the present study. Modulation of cytokine responsive pathways like JAK/STAT may contribute to the observations in myoblast proliferation and enhanced differentiation, since this pathway regulates the balance between these stages of myogenesis (Diao et al. 2009; Hoene et al. 2013; Sun et al. 2007; Trenerry et al. 2011; Wang et al. 2008). This may occur through downstream effects on myogenic regulatory factors, including MyoD, which play a role in coordinating myoblast differentiation and fusion (Rudnicki and Jaenisch 1995; Tsuji et al. 2025; Yang et al. 2009). Tomatidine's ability to modulate JAK/STAT signaling in models of human skeletal muscle supports this contention (Parafati et al. 2025). In parallel, mTORC1 signaling represents a well‐established regulator of muscle hypertrophy (Bodine 2022), protein synthesis (Morita et al. 2015) and myotube formation (Kikani et al. 2019; Park and Chen 2005; von Walden et al. 2016) and has been identified previously as a target of tomatidine in mature skeletal muscle (Dyle et al. 2014). The transient increase in protein synthesis during early differentiation in the present study suggests tomatidine may activate anabolic signaling pathways during a critical window of myotube formation, thereby contributing to the enhanced hypertrophic response. Although tomatidine did not alter the phosphorylation of STAT3, p70S6K, S6 or 4EBP1 at the time points examined, this does not necessarily rule out the involvement of JAK/STAT or mTORC1 signaling. Phosphorylation events of these pathways are highly transient, and it is possible that tomatidine‐induced changes occurred outside the sampling window.

Taken together, these pathways provide plausible mechanistic explanations for the effects of tomatidine observed in the present study. It should be noted that these experiments were designed to phenotypically characterize tomatidine's effects on myoblast proliferation, differentiation, fusion, and hypertrophy, rather than directly assess the activity of specific signaling pathways. Consequently, the potential involvement of JAK/STAT, mTORC1, and other signaling networks should be considered provisional and based on current literature. Future mechanistic studies will be required to determine the relative contribution of these pathways to the pro‐myogenic effects of tomatidine.

## 5 Conclusions

Collectively, the findings of this study demonstrate that tomatidine exerts stage‐specific effects on myogenic progression, influencing multiple phases of muscle cell development. While tomatidine suppresses myoblast proliferation, it enhances early differentiation, promotes myoblast fusion, and increases myotube hypertrophy, supported by a transient elevation in protein synthesis during a critical window of myotube formation. These coordinated effects suggest tomatidine facilitates a shift from proliferative expansion toward a pro‐differentiation, anabolic state, ultimately driving the formation of fewer, larger myotubes with greater myonuclei content. These effects could be harnessed for applications optimizing muscle cell development and growth, especially in the context of cellular agriculture for the production of cultivated meat and seafood. In the context of muscle regeneration, such effects may be beneficial by enhancing the efficiency of myofiber repair and growth, particularly when appropriately timed. However, the stage‐dependent effects observed in this study highlight the importance of optimizing the timing and context of intervention to maximize regenerative outcomes.

## Author Contributions

**Marissa K. Caldow:** conceptualization, methodology, writing, formal analysis, investigation, writing – original draft, writing – review and editing, visualization, project administration. **Kristy Swiderski:** conceptualization, methodology, writing, formal analysis, investigation, writing – original draft, visualization. **Emily S. Haber:** investigation, writing – review and editing. **John H. V. Nguyen:** investigation, writing – review and editing. **Kyla J. H. Fritz:** investigation, writing – review and editing. **Gordon S. Lynch:** conceptualization, methodology, writing – original draft, writing ‐ review and editing, supervision.

## Ethics Statement

This study did not involve experiments with human participants or animals. All experiments were performed using cell lines; murine C2C12 myoblasts (ATCC).

## Conflicts of Interest

The authors declare no conflicts of interest.

## Supporting information


**Figure S1:** Tomatidine supplementation during differentiation of muscle cells. C2C12 cells were differentiated in standard differentiation media containing vehicle control (DMSO, A) or media supplemented with 10 µM tomatidine (TOM, B) from the onset of differentiation, for up to 4 days. To determine effects of tomatidine on differentiation with a shorter treatment, differentiating myoblasts were supplemented with tomatidine for the first 2 days of differentiation only (D0‐D2; TOM > DMSO, C), then returned to vehicle supplemented differentiation media for the following 2 days. The treatment protocol was also inverted to day 2‐4 of differentiation (D2‐D4, DMSO > TOM, D), with cells differentiated in DMSO for the first 2 days of differentiation, then swapped to tomatidine supplementation for the next 2 days. DMSO, vehicle treated control cells. TOM, cells supplemented with 10 µM tomatidine. Scale bar represents 50 µm.
**Figure S2:** Acute tomatidine supplementation promotes increased protein synthesis. C2C12 cells were differentiated in standard differentiation media containing vehicle control (DMSO) or media supplemented with 10 µM tomatidine from the onset of differentiation. Puromycin protein abundance was measured 1hour after the onset of differentiation with TOM supplementation. Data are presented as mean ± SD (n = 3 experiments), normalized to DMSO. Significantly different to DMSO, ***P < 0.001. GM, growth media before differentiation media was applied. DMSO, vehicle treated control cells. TOM, cells supplemented with 10 µM tomatidine.
**Figure S3:** Tomatidine supplementation does not alter STAT3, or mTORC1 related signaling during early myogenesis. C2C12 cells were differentiated in standard differentiation media containing vehicle control (DMSO) or media supplemented with 10 µM tomatidine from the onset of differentiation. Phosphorylation status of STAT3 (Tyr705) (A), p70S6K (Thr 389), S6 (Ser 240/244) and 4EBP1 (Ser65) (B) were measured 1, 48, 49 and 96 hours after the onset of differentiation with TOM supplementation. Samples for 1 and 49 hours were collected following application/refreshment of differentiation media. Data are presented as mean ± SD (n = 3 experiments), normalized to DMSO. GM, growth media before differentiation media was applied. DMSO, vehicle treated control cells. TOM, cells supplemented with 10 µM tomatidine.

**Video S1:** C2C12 cells were differentiated for 4 days in standard differentiation media containing vehicle control (DMSO) or media supplemented with 10 µM tomatidine from the onset of differentiation. Live cell imaging was performed from D2‐D4 of differentiation. Images were captured each hour, across 45 hours. Video represents cells differentiated for 4 days in standard differentiation media containing vehicle control (DMSO).

**Video S2:** C2C12 cells were differentiated for 4 days in standard differentiation media containing vehicle control (DMSO) or media supplemented with 10 µM tomatidine from the onset of differentiation. Live cell imaging was performed from D2‐D4 of differentiation. Images were captured each hour, across 45 hours. Video represents cells differentiated for 4 days in standard differentiation media supplemented with 10 µM tomatidine.

## Data Availability

The datasets used and/or analyzed during the current study are available from the corresponding author on reasonable request.
