# Supplementary material for: Tomatidine Exposure During Early Myogenesis Promotes Hypertrophy of C2C12 Muscle Cells Through Increased Nuclei Accretion
Source: Cell Biol Int. 2026 Oct 8;50(10):e70224. doi: 10.1002/cbin.70224 (PMC13648157; doi:10.1002/cbin.70224)
Supplement: Supplementary file 1 [file CBIN-50-0-s001.docx]

**Appendix A: Supplemental data**

**Fig. S1:** Tomatidine supplementation during differentiation.

*(Daily brightfield images from D0-D4)*

**Fig. S2:** Acute tomatidine supplementation promotes increased protein synthesis

*(Puromycin abundance 1 hr after TOM exposure)*

**Fig. S3:** Tomatidine supplementation does not alter STAT3, or mTORC1 related signaling during early myogenesis

*(STAT3, P70S6K, S6 and 4EBP1 immunoblotting)*

**Supp. Video 1:** Live cell imaging-DMSO

*(video file, and still frame)*

**Supp. Video 2:** Live cell imaging-TOM

*(video file, and still frame)*

**Appendix A: Supplementary Material**

**Tomatidine exposure during early myogenesis promotes hypertrophy of C2C12 muscle cells through increased nuclei accretion**

Marissa K. Caldow, Kristy Swiderski, John H. V. Nguyen, Emily S. Haber, Kyla J. H. Fritz, Gordon S. Lynch*

**Fig S1: Tomatidine supplementation during differentiation of muscle cells.** C2C12 cells were differentiated in standard differentiation media containing vehicle control (DMSO, **A**) or media supplemented with 10 µM tomatidine (TOM, **B**) from the onset of differentiation, for up to 4 days. To determine effects of tomatidine on differentiation with a shorter treatment, differentiating myoblasts were supplemented with tomatidine for the first 2 days of differentiation only (D0-D2; TOM > DMSO, **C**), then returned to vehicle supplemented differentiation media for the following 2 days. The treatment protocol was also inverted to day 2-4 of differentiation (D2-D4, DMSO > TOM, **D**), with cells differentiated in DMSO for the first 2 days of differentiation, then swapped to tomatidine supplementation for the next 2 days. DMSO, vehicle treated control cells. TOM, cells supplemented with 10 µM tomatidine. Scale bar represents 50 µm.

**Fig S2: Acute tomatidine supplementation promotes increased protein synthesis.** C2C12 cells were differentiated in standard differentiation media containing vehicle control (DMSO) or media supplemented with 10 µM tomatidine from the onset of differentiation. Puromycin protein abundance was measured 1hour after the onset of differentiation with TOM supplementation. Data are presented as mean ± SD (n = 3 experiments), normalized to DMSO. Significantly different to DMSO, ****P* < 0.001. GM, growth media before differentiation media was applied. DMSO, vehicle treated control cells. TOM, cells supplemented with 10 µM tomatidine.

**Fig S3: Tomatidine supplementation does not alter STAT3, or mTORC1 related signaling during early myogenesis.** C2C12 cells were differentiated in standard differentiation media containing vehicle control (DMSO) or media supplemented with 10 µM tomatidine from the onset of differentiation. Phosphorylation status of STAT3 (Tyr705) (**A**), p70S6K (Thr 389), S6 (Ser 240/244) and 4EBP1 (Ser65) (**B**) were measured 1, 48, 49 and 96 hours after the onset of differentiation with TOM supplementation. Samples for 1 and 49 hours were collected following application/refreshment of differentiation media. Data are presented as mean ± SD (n = 3 experiments), normalized to DMSO. GM, growth media before differentiation media was applied. DMSO, vehicle treated control cells. TOM, cells supplemented with 10 µM tomatidine.

**Video S1:** C2C12 cells were differentiated for 4 days in standard differentiation media containing vehicle control (DMSO) or media supplemented with 10 µM tomatidine from the onset of differentiation. Live cell imaging was performed from D2-D4 of differentiation. Images were captured each hour, across 45 hours. Video represents cells differentiated for 4 days in standard differentiation media containing vehicle control (DMSO).

(still frame for video placeholder, DMSO 20 h, scale bar represents 50 µm)

**Video S2:** C2C12 cells were differentiated for 4 days in standard differentiation media containing vehicle control (DMSO) or media supplemented with 10 µM tomatidine from the onset of differentiation. Live cell imaging was performed from D2-D4 of differentiation. Images were captured each hour, across 45 hours. Video represents cells differentiated for 4 days in standard differentiation media supplemented with 10 µM tomatidine.

(still frame for video placeholder, tomatidine 20 h, scale bar represents 50 µm)
